# EXPERIENTIAL ASSESSMENT OF AN INNOVATIVE DEMENTIA CARE TRAINING SEQUENCE FOR HEALTH AND HUMAN SERVICES WORKERS

**DOI:** 10.1093/geroni/igac059.2485

**Published:** 2022-12-20

**Authors:** Susan Wehry, David Wihry

**Affiliations:** University of New England, Biddeford, Maine, United States; University of Maine, Orono, Maine, United States

## Abstract

This poster presents results of an assessment of an interdisciplinary dementia care training titled Dementia Reconsidered (DR). In contrast to disease and deficit-focused training curricula, DR incorporates and emphasizes the strengths and humanity of people living with dementia and focuses on person-directed support for, and care of, persons living with dementia. For example, DR makes the case for an approach to shared and supported decision-making that optimizes the individual’s— and significant others’—abilities. The on-line lectures were chunked to accommodate cognitive load and active learning; 20-30 second stretch breaks were interspersed to facilitate attention. This may account for the higher retention and utilization rates of DR than are often found in one-off trainings. A retrospective pre-post survey administered at the conclusion of DR sessions measured participant learning outcomes. Participants in DR sessions were representative of the fields of social work (42%), nursing (21%), and a significant “other” category (28%), mostly long-term care administrators. Among post-training survey respondents (N=36), 77% improved their knowledge of the key principles of person-directed dementia care; 73% (N=37) showed improved comfort in supporting residents with dementia during the CoVID-19 pandemic; 83% (N=24) showed improvement in ability to distinguish capacity and competency; and 83% (N=23) reported increased comfort in supporting people with dementia in making decisions in the face of diminished capacity. At six month follow-up, 63% (N=33) had used a skill gained from their training (17% no, 20% not sure). Implications for training in dementia care practices will be discussed.

